# Differences in game-based performance by playing position in young elite male team handball players

**DOI:** 10.3389/fspor.2025.1688078

**Published:** 2025-10-16

**Authors:** Herbert Wagner, Vanja Radic, Matthias Hinz

**Affiliations:** ^1^ Department of Sport and Exercise Science, University of Salzburg, Salzburg, Austria; ^2^ Department of Sport Science, Otto von Guericke University of Magdeburg, Magdeburg, Germany

**Keywords:** physical performance, oxygen uptake, agility, jump shot, offence, defense, sprinting

## Abstract

**Introduction:**

In male team handball, different playing positions have different demands due to their tactical roles. However, if the game-based physical performance differs across playing positions has not been analyzed, although this is crucial for training young elite players to reach a world-class level based on their specific positions. Consequently, the aim of this study was to analyze game-based performance in young elite male team handball players based on their playing positions.

**Methods:**

Forty-eight young elite male team handball players (age: 17.5 ± 1.9 years, body weight: 82.5 ± 9.9 kg, body height: 1.86 ± 0.05 m), including 23 backs, 17 wings and 8 pivots participated in the study. All players trained 7–8 sessions per week at an elite team handball academy and competed at the highest international level for their age group. To determine specific physical performance, all participants performed the team handball game-based performance test. A one-way ANOVA was used to compare the performance differences among backs, wings and pivots.

**Results:**

Significant differences between playing positions (*P* < 0.05) were found in peak oxygen uptake, heart rate, fast break and offense time, jump height during the jump shot, and body mass. Wings showed the best performance in fast break (1.78 ± 0.08 s), offense time (5.74 ± 0.19 s), jump height during the jump shot (0.39 ± 0.06 m), and peak oxygen uptake (72.4 ± 8.4 ml/kg/min). Backs performed best in ball velocity during the jump shot (25.1 ± 1.5 m/s), while pivots had the highest body weight (90.5 ± 14.1 kg).

**Discussion:**

As expected, pivots were the heaviest due to facing the most physical contact with defenders during matches. Wings were the fastest on the court and jumped the highest, while backs demonstrated the highest throwing velocities, as they frequently perform long-distance throws during games. However, the high levels of peak oxygen uptake for wings and backs (around 70 ml/kg/min) and pivots (around 60 ml/kg/min), along with no significant differences in defense time between positions, highlight the importance of both aerobic and anaerobic performance for all players to maintain an active and dynamic performance throughout the entire match.

## 1 Introduction

Team handball is a fast-paced, high-intensity sport played indoors by two teams of seven players (six field players and one goalkeeper). The game emphasizes rapid transitions, frequent physical contact, and complex technical and tactical execution. Matches consist of two 30-minute halves and involve continuous movement, sprinting, jumping, and powerful throwing actions. Due to its dynamic nature, team handball places high physical, technical, and tactical demands on players. Performance is influenced by position-specific roles, each requiring different levels of speed, strength, endurance, and decision-making skills (1, 2). Understanding these multifaceted demands is essential for effective player development, training optimization, and injury prevention in both amateur and elite settings. Goalkeepers rely on quick reflexes, agility, and anticipation to defend the goal. Wing players (left and right), positioned near the sidelines, are typically fast and agile, excelling in quick counterattacks and sharp-angle shots. Backcourt players (left, center, right) serve as primary shooters and playmakers, requiring high ball velocity, tactical vision, and one-on-one skills. Pivots (line players) operate around the 6-meter line, engaging defenders to create space for teammates. They require strength, balance, and precise positioning to catch and shoot under defensive pressure.

In previous studies, positional differences in team handball have been examined either through activity profiles during matches, using camera tracking or local positioning systems, or by assessing physical demands with tests of strength, power, endurance, sprinting, and jumping abilities. When comparing the different field player positions, pivots had the highest body weight, while wing players (wings) had the lowest body weight and height but demonstrated faster sprinting and higher maximal oxygen uptake. Backcourt players (backs) achieved the highest ball velocity (3–8). Match analysis showed that wings had longer playing time, covered greater distances, and tended to perform more sprints and high-intensity runs (1, 2, 9–16). Differences in player load and the number of high-intensity actions were also observed between playing positions, as well as between offensive specialists and two-way players who participate in both offense and defense (17, 18). However, whether specific game-based physical performance differs between playing positions has not been analyzed in previous studies. This gap is critical, as understanding such differences is essential for position-specific training in elite team handball, particularly for developing young elite players to reach world-class performance levels tailored to their playing roles.

To determine the specific physical performance we choose the team handball game-based performance test (GBPT) because it is a sport-specific and validated test (19) that comprehensively measures the physical performance demands of team handball. The GBPT is uniquely suited to assess key performance components such as agility (offense and defense, including accelerations, decelerations, and changes of direction), sprinting (fast breaks and fast retreats), throwing and jumping (during jump shots), and aerobic capacity (via peak oxygen uptake). Furthermore, previous research has demonstrated the test's ability to differentiate performance based on factors such as performance level (20, 21), sex (22), and age (23). However, since differences between playing positions have not yet been explored, the GBPT provides an ideal framework for addressing this gap in the literature while ensuring the validity and relevance of our findings.

Consequently, the aim of this study was to analyze game-based physical performance in young elite male team handball players based on their playing positions. We hypothesized that sprinting performance, throwing and jumping performance during the jump shot, as well as body mass, would differ between wings, backs, and pivots.

## 2 Methods

### 2.1 Participants

Forty-eight young elite male team handball players (age: 17.5 ± 1.9 years, body mass: 82.5 ± 9.9 kg, height: 1.86 ± 0.05 m) participated in the study, including 23 backs (7 left, 7 right, 9 center), 17 wings (9 left, 8 right), and 8 pivots. Fourteen participants were left-handed and 34 right-handed. Goalkeepers were excluded, as the game-based performance test was designed specifically for field players. All athletes trained 7–8 sessions per week (approximately 10–12 h) at an elite handball academy and competed at the highest international level for their age group. All participants were healthy, physically fit, and injury-free at the time of the study. The study was approved by the Ethics Committee of the University of Salzburg (GZ-44-2015) in accordance with the Declaration of Helsinki. Written informed consent was obtained from all participants; for minors, consent was also provided by their legal guardians.

### 2.2 Procedures

The study employed a cross-sectional comparative design to examine differences in specific physical performance across handball playing positions. To ensure standardized testing conditions, all tests were conducted over four consecutive days within a single week, specifically during the fourth week of the pre-season preparation phase. This period was selected to reflect peak specific physical performance, assuming optimal training adaptation had occurred during the earlier weeks of preparation.

Before starting the GBPT, all players received a theoretical familiarization, during which the test administrator explained the procedures in detail. To ensure optimal test efficiency, players were divided into groups of at least four, preferably with similar playing positions and handedness. In each group, one player performed the test, two served as passers, and the fourth prepared for the next trial. The warm-up included 20 min of general and sport-specific exercises, replicating typical training and competition activities. Following the warm-up, measuring equipment was attached to the test subject, and all systems were calibrated and activated simultaneously. A two-minute countdown preceded a practical familiarization run of the GBPT, consisting of submaximal movements (approximately 70% of maximum effort) to help players adapt to the test environment.

**Figure 1:**
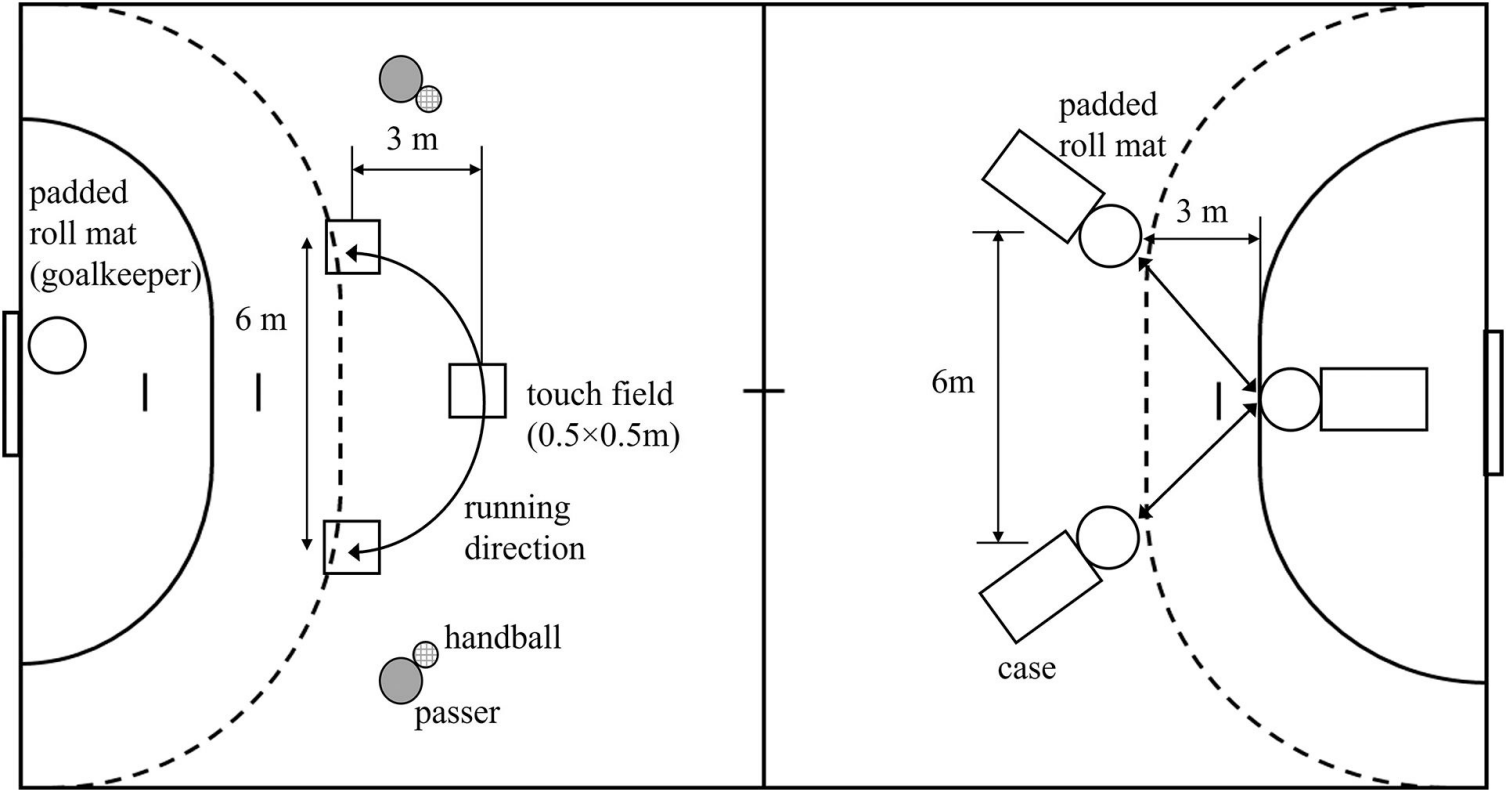
Schematic figure of the team handball game-based performance test.

All participants completed eight repetitions (heats) of the GBPT, which included handball-specific movements such as offensive and defensive actions, transitions between offense and defense, and active recovery periods. During defensive tasks, players were required to sprint from the 6 m line to the 9 m line and simulate tackles by touching padded roll mats (Figure 1) with both hands. Right-handed players touched the left roll mat twice and the right mat once, while left-handed players followed the opposite sequence. During offensive actions, players sprinted from the 12 m line to the 9 m line, contacting square target zones (0.5 × 0.5 m) marked on the floor (Figure 1) using at least one foot. Simultaneously, they had to catch and pass the ball. Right-handed players touched the right target zone twice and the left once, while left-handed players performed the mirrored sequence. All running distances were standardized using the positions of the target zones and padded roll mats, ensuring consistency across participants regardless of handedness. In heats #2, #3, #4, #6, and #8, participants were required to conclude the offensive sequence with a jump shot. During the jump shot, right-handed players performed a maximal take-off from the left foot on the right marked zone, aiming to throw the ball with maximum velocity into the lower left corner of the goal. In contrast, left-handed players executed the mirror movement pattern, taking off from the right foot on the left marked zone and aiming at the lower right corner of the goal. In heats #3 and #6, participants initiated fast breaks by sprinting from defense to offense. This began after touching the padded roll mat at the 6 m line, followed by receiving a pass at the 12 m line, and concluding the sequence with a jump shot from 9 m in offense. During the fast break, right-handed players performed take-off on the right marked area, while left-handed players followed the opposite pattern. In heats #4 and #6, participants also executed fast retreats, transitioning immediately from offense to defense after completing the jump shot.

### 2.3 Measurements

To measure defense time (from the first to the last contact on the padded roll mats), offense time (from the first contact on the square marked areas), 10 m fast break time (from the 9 m line in defense to the middle line), and 20 m fast break time (from the middle line to the square marked areas in offense), three hand stopwatches (Hanhart Stratos 2, Hanhart GmbH, Gütenbach, Germany) were used. However, due to differing accelerations after the jump shot, fast retreat time was excluded from subsequent evaluations. Break durations, including those between offense and defense actions (20 s), between two defensive or offensive actions (15 s), and between two heats (40 s), were monitored using the Multi-Timer-Ultimate software (Multi-Timer-Ultimate 3.1, Wallroth, Berlin, Germany). Breaks commenced immediately following the completion of offensive or defensive actions, with the last three seconds audibly counted down (“3, 2, 1, go”). To determine ball velocity and jump height during the jump shots, all jump shots were recorded using a high-speed video camera (JVC-GC-PX100BE, JVC, Yokohama, Japan) at 200 frames per second. The Tracker Video Analysing Software (Tracker 4.59, Douglas Brown, Aptos, California, US) was then used to analyze the 2D position of the ball's center and calculate flight time. Calibration was done using a team handball goal (4-point calibration, 2 × 3 m). Flight time was defined as the duration between the last floor contact of the take-off foot and the first contact of the landing foot. Ball velocity was determined by analyzing the linear velocity of the ball's 2D position after release (over 15 frames). Jump height was calculated using the equation below, where g is gravitational acceleration. The mean values of the best three attempts were used for analysis and evaluation.

$$h_{jump}=\frac{g}{8}\times t_{flight}^{2}$$

During the GBPT, heart rate and oxygen uptake were measured using a heart rate belt with a sensor module (Suunto T6d, Suunto, Vantaa, Finland) and a portable breath-by-breath gas analysis system (K5, Cosmed, Rome, Italy). Peak heart rate (HRpeak) and peak oxygen uptake (VO₂peak) values were calculated across all heats of the GBPT for subsequent analysis. To ensure the accuracy of VO₂peak determination, only those peak values were considered where the two consecutive breath-by-breath measurements immediately before and after the peak were not less than 90% of the peak value.

### 2.4 Statistical analysis

All statistical analyses were conducted using SPSS version 29.0 (SPSS Inc.). Means, standard deviations, and 95% confidence intervals were calculated for descriptive statistics. The normality of the variables was confirmed using the Shapiro–Wilk test. To assess differences between the playing positions, a one-way ANOVA was employed. When significant effects were found, a Bonferroni *post hoc* test was used to identify differences between backs, wings, and pivots. Effect size (η^2^) and statistical power (1-β) were also calculated. Statistical significance was set at *p* < .05. Effect sizes were interpreted as small (0.01 ≤ η^2^ < 0.06), medium (0.06 ≤ η^2^ < 0.14), and large (η^2^ ≥ 0.14).

## 3 Results

Table 1 presents the mean values, standard deviations, 95% confidence intervals, F-values, *P*-values, and effect sizes from the one-way ANOVA, along with *post hoc P*-values for VO_2_peak, HRpeak, 10 m and 20 m fast break times, defensive and offensive times, jump height and ball velocity in the jump shot, body mass, and body height during the GBPT. Significant differences were found in VO_2_peak, HRpeak, 10 m and 20 m fast break times, offensive time, jump height, and body mass (Table 1). Wings and backs showed a 19%–24% higher VO_2_peak than pivots. Wings had a 5% faster 10 m sprint time and backs a 7% faster 20 m sprint time than pivots. Wings and backs also completed offensive actions 6%–7% faster than pivots. Additionally, wings demonstrated a 30% higher jump height in the jump shot, while pivots had a 16% higher body mass than wings.

**Table 1:** Mean values, standard deviation (SD), 95% confidence intervals (95% CI), *F*-values, *P*-values and *effect size* of the One-way ANOVO, *P*-values of the *post hoc*-tests in the peak oxygen uptake (VO_2_peak), peak heart rate (HRpeak), 10 m- and 20 m-fast break time, defensive and offensive time, jump height and ball velocity in the jump shot, body mass and body height in the team handball game-based performance test.

Measured variables	Backs mean ± SD (95% CI)	Wings mean ± SD (95% CI)	Pivots mean ± SD (95% CI)	ANOVA F-, *P*-value *Effect size*	*Post hoc*-Tests Positions *P*-value	
VO_2_peak (ml/kg.min)	69.5 ± 8.3 (65.9–73.0)	72.4 ± 8.3 (68.1–76.7)	58.3 ± 7.8 (51.7–64.8)	8.16, <0.01** 0.27	Backs-Pivots <0.01**	Wings-Pivots <0.001***
HRpeak (bpm)	192 ± 6 (189–194)	187 ± 8 (183–191)	196 ± 10 (188–204)	4.75, 0.02* 0.17		
10 m-fast break time (s)	1.82 ± 0.08 (1.78–1.85)	1.78 ± 0.08 (1.74–1.83)	1.89 ± 0.12 (1.78–1.99)	3.42, 0.04* 0.13		Wings-Pivots 0.02*
20 m-fast break time (s)	1.76 ± 0.11 (1.71–1.81)	1.78 ± 0.12 (1.72–1.84)	1.90 ± 0.16 (1.77–2.03)	4.10, 0.02* 0.15	Backs-Pivots 0.03*	
Defense time (s)	5.53 ± 0.18 (5.46–5.61)	5.44 ± 0.22 (5.32–5.55)	5.63 ± 0.29 (5.39–5.87)	2.37, 0.11 0.10		
Offence time (s)	5.81 ± 0.27 (5.69–5.92)	5.74 ± 0.19 (5.65–5.84)	6.19 ± 0.28 (5.96–6.42)	9.71, <0.001*** 0.30	Backs-Pivots <0.01**	Wings-Pivots <0.001***
Jump height (m)	0.36 ± 0.07 (0.33–0.39)	0.39 ± 0.06 (0.36–0.43)	0.30 ± 0.05 (0.26–0.34)	5.46, <0.01** 0.20		Wings-Pivots <0.01**
Ball velocity (m/s)	25.1 ± 1.5 (24.5–25.8)	24.4 ± 1.5 (23.6–25.2)	24.1 ± 1.5 (22.9–25.3)	1.74, 0.19 0.07		
Body mass (kg)	82.8 ± 5.8 (80.3–85.3)	78.3 ± 10.2 (73.1–83.5)	90.5 ± 14.1 (78.7–102.3)	4.85, 0.01* 0.18		Wings-Pivots 0.01*
Body height (m)	1.85 ± 0.05 (1.83–1.88)	1.85 ± 0.06 (1.82–1.88)	1.89 ± 0.04 (1.85–1.92)	1.29, 0.29 0.05		

*P < 0.05, **P < 0.01, ***P < 0.001.

**Figure 2:**
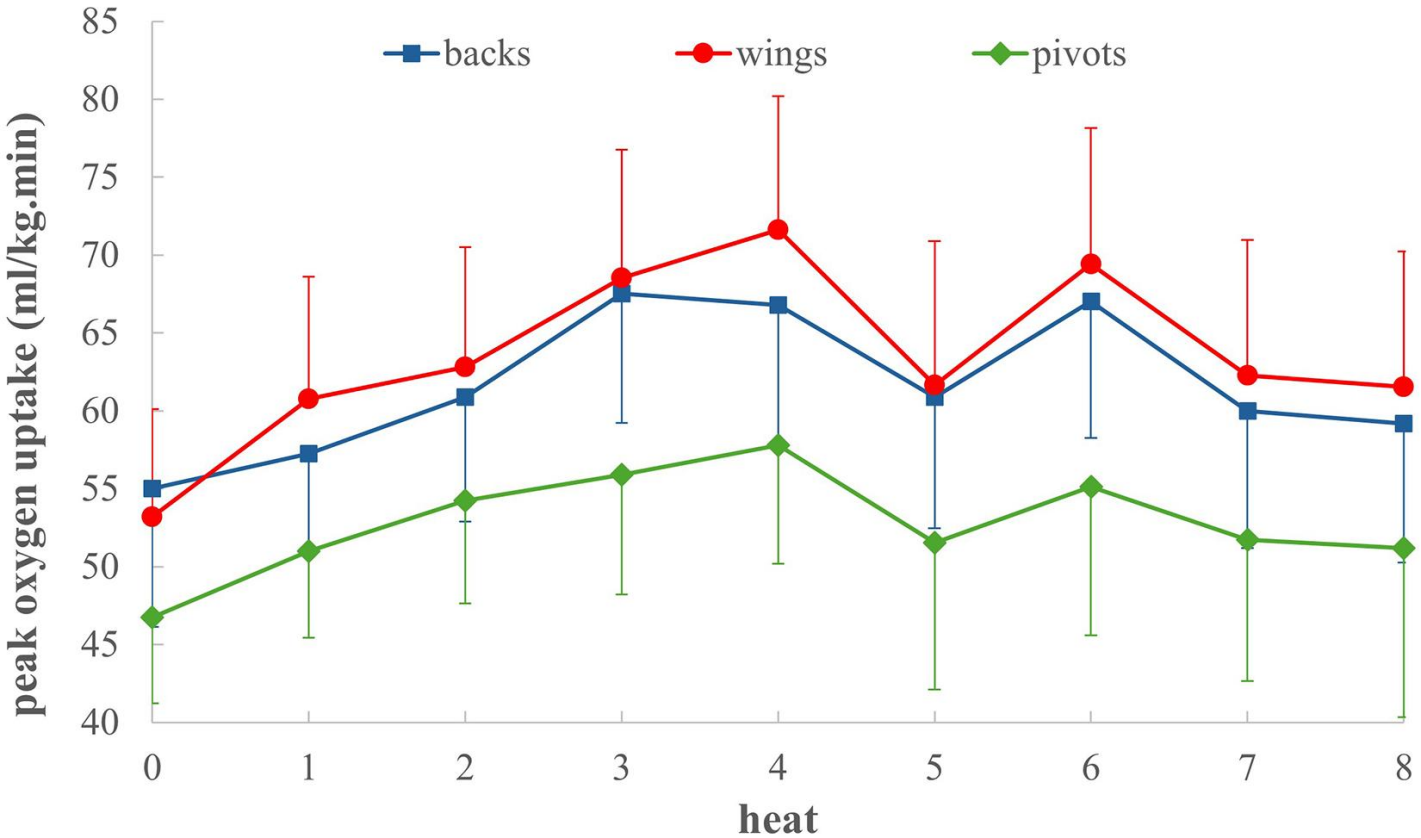
Mean values (± standard deviations) in the peak oxygen uptake of the backs, wings and pivots, separated in the different heats in the team handball game-based performance test.

**Figure 3:**
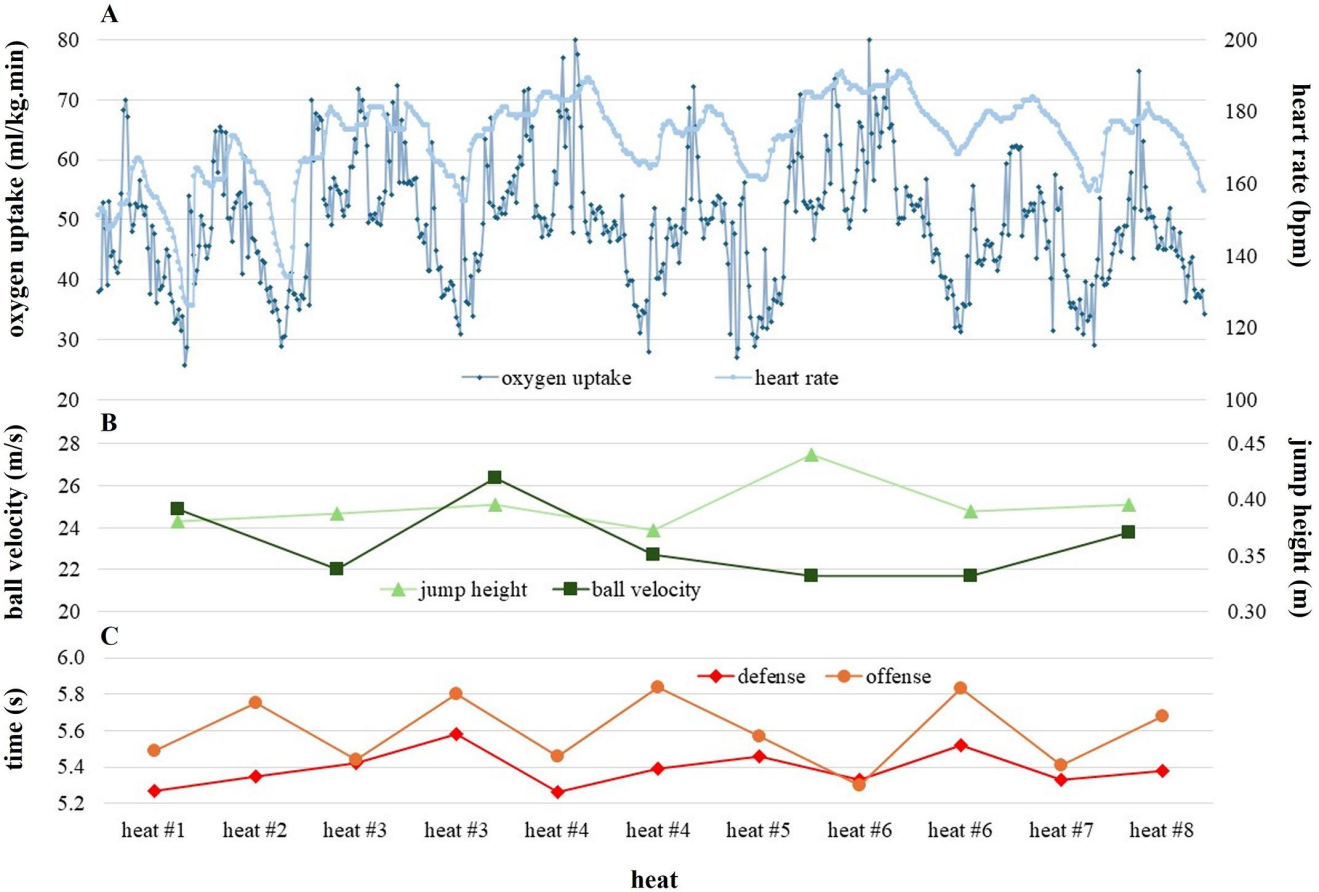
Detailed data from a 17.2-year-old center back in heart rate and breath-by-breath response in oxygen uptake **(A)**, ball velocity and jump height across the seven analyzed jump shots **(B)**, as well as offensive and defensive time **(C)** in heat #1–8 in the team handball game-based performance test.

Figure 2 presents the mean (± standard deviation) VO_2_peak values for backs, wings, and pivots across the different heats of the GBPT. A noticeable increase in VO_2_peak is observed in heats #3, #4, and #6 for all positions, with wings and backs consistently reaching higher VO_2_peak values than pivots in each heat. Figure 3 illustrates detailed data from a 17.2-year-old center back as a representative example. In Figure 3A, the increase/decrease in heart rate at the start and end of each heat, along with the rapid breath-by-breath response in oxygen uptake, are clearly visible. Figure 3B highlights the intraindividual variability in ball velocity and jump height across seven analyzed jump shots. Figure 3C shows a distinct increase in offensive time during the second offensive sequence in heats #3, #4, and #6.

## 4 Discussion

As expected, we found significant differences between playing positions in sprinting time, jump height during the jump shot, and body weight. Wings demonstrated faster acceleration than pivots in the 10 m-fast break time (1.78 ± 0.08 s vs. 1.89 ± 0.12 s), while backs outperformed pivots in the 20 m-fast break time (1.76 ± 0.11 s vs. 1.90 ± 0.16 s). In team handball, wings are typically the first players to initiate fast breaks, whereas pivots often play in central defensive roles. We suggest that the superior 10 m-fast break time of wings reflects their greater familiarity with rapid acceleration in fast break situations during the game. Backs, on the other hand, are frequently responsible for receiving passes from the goalkeeper and executing jump shots in transitional play during the game. This likely explains their advantage in the 20 m-fast break time during the GBPT. Our findings are consistent with previous research reporting superior sprint performance among wings and a higher frequency of sprints and high-intensity runs during matches (1–8, 12–15, 24). The significant difference in jump height between wings and pivots in the GBPT is likely related to the different demands of their playing positions. Wing players commonly use jump shots during matches, while pivots often shoot under physical pressure from defenders. As the GBPT involves a jump shot without contact, this may explain the better performance of wings in this test (4, 6). Although no significant differences were found in ball velocity, backs showed higher values compared to wings and pivots, with a medium effect size (η^2^ = 0.07). This is likely due to the typical in-game role of backs, who often shoot from distances greater than 8 meters and therefore require higher ball velocity to score. Higher ball velocities in the jump shot in backs, compared to wings and pivots were also found in previous studies (3, 25). The higher body weight of pivots compared to wings and backs may be explained by their specific role in the game, which involves frequent physical contact, blocking opposing defenders, and shooting under pressure. This positional characteristic is consistent with previous findings (3, 6, 26).

We found significant differences in sprinting performance between playing positions, but not in defense time. In modern elite team handball, defensive play has become highly dynamic, requiring rapid movement, frequent changes of direction, and quick acceleration from all positions. Players must win one-on-one duels, execute stop-fouls, and provide immediate support when a teammate loses a defensive contest. These demands apply equally to wing defenders, who are responsible for closing down the wide areas, and to central defenders, who must cover a larger defensive zone and provide support across the court. Pivots, although their primary offensive role is different to wings and backs, often play as central defenders and are therefore repeatedly required to move quickly laterally and forwards to block breakthroughs. This universal need for speed and agility in defense may explain the lack of positional differences in defensive time despite clear differences in sprinting ability. In contrast to the findings for defensive performance, we observed significant differences in offense time between pivots (6.19 ± 0.28 s), wings (5.74 ± 0.19 s), and backs (5.81 ± 0.27 s). These differences are likely related to the distinct offensive roles of each position during match play. In competition, wings and backs frequently perform actions closely resembling the demands of the GBPT, including rapid sprints, frequent changes of direction, and coordinated ball handling through catching and passing. Pivots, in contrast, typically operate near the 6 m line, engaging in physical duels with defenders and performing short explosive movements rather than extended sprinting or multi-directional runs. This positional specificity may explain their longer offense times in the GBPT. Notably, the offense and defense times of the wings and backs in the present study, aside from the pivots, were considerably better than those reported for adult male players competing in the Austrian first league and the German third and fourth leagues (21, 23), highlighting the high physical and technical level of the adolescent elite players investigated in our study.

Significant differences between playing positions were also observed in VO₂peak during the GBPT. Wings (72.4 ± 8.2 ml·kg^−^^1^·min^−^^1^) and backs (69.5 ± 8.3 ml·kg^−^^1^·min^−^^1^) demonstrated higher aerobic capacity compared to pivots (58.3 ± 7.8 ml·kg^−^^1^·min^−^^1^). Two main factors may explain these differences. First, VO₂peak values were calculated relative to body weight; therefore, the higher body weight of pivots naturally reduces their relative VO₂peak. Second, positional demands differ markedly in match play. Wings and backs typically execute more rapid sprints, frequent changes of direction, and repeated high-intensity actions in offense, which place greater demands on both anaerobic and aerobic systems. Enhanced aerobic capacity is likely advantageous for these positions, allowing for faster recovery during short breaks and sustaining high-intensity efforts throughout the game. This pattern was also evident in the GBPT, as shown in Figure 2, where higher VO₂peak values occurred in heats #3, #4, and #6 involving multiple defensive and offensive sequences combined with fast breaks and retreats. Furthermore, the rapid breath-by-breath VO₂ responses observed across all heats as shown in Figure 3 highlight the ability of wings and backs to quickly meet increased energy demands. Notably, the VO₂peak values for wings and backs in this study exceeded those reported for adult male players in the Austrian first league and German third and fourth leagues (21, 23), emphasizing the exceptional aerobic fitness of these adolescent elite athletes. Overall, these findings underline the importance of both aerobic and anaerobic performance capacities for maintaining high-intensity, dynamic play across all positions in modern team handball.

A limitation of the study was that the offensive actions in the GBPT more closely resembled the game-specific movements of wings and backs than those of pivots, as previously discussed. For the sake of test standardization, however, it was necessary to apply identical offensive and defensive tasks across all participants. Another limitation was the use of a portable breath-by-breath gas analysis system. The setup consisted of a rubber face mask connected to the gas analyzer via a plastic tube, with the device fixed on the back using a belt system. While the face mask may have influenced breathing and the belt system may have minimally affected movements such as throwing and sprinting, the impact was likely small. Participants were accustomed to spiroergometry testing, and after the warm-up heat, they appeared adapted to the equipment, suggesting that it did not meaningfully hinder performance during the GBPT.

In summary, this study demonstrated that offensive performance variables in the GBPT, including offensive time, sprinting, jumping, and throwing performance, as well as aerobic capacity, are closely related to playing position in young male elite handball players, whereas defensive performance was relatively similar across positions. Overall, the players in this study showed markedly higher performance levels compared to adult athletes from the Austrian first league and the German third and fourth leagues, underlining their highly competitive standard. The comparable performance of wings and backs in the GBPT suggests that transitions between these positions may be feasible later in a career, while pivots, due to lower sprinting, jumping, and throwing performance, may face greater limitations in switching to wing or back positions.

## Data Availability

The original contributions presented in the study are included in the article/Supplementary Material, further inquiries can be directed to the corresponding author.
